# Approach to the patient with controlled acromegaly and acromegalic arthropathy: clinical diagnosis and management

**DOI:** 10.1007/s11102-024-01465-1

**Published:** 2024-11-01

**Authors:** Iris C. M. Pelsma, Herman M. Kroon, Cornelie D. Andela, Enrike M. J. van der Linden, Margreet Kloppenburg, Nienke R. Biermasz, Kim M. J. A. Claessen

**Affiliations:** 1 Department of Medicine, Division of Endocrinology, and Center for Endocrine Tumors Leiden, Leiden University Medical Center, Albinusdreef 2, 2333 ZA, Leiden, The Netherlands; 2 Department of Radiology, Leiden University Medical Center, Leiden, The Netherlands; 3 Basalt Rehabilitation Center, The Hague, The Netherlands; 4 Department of Orthopedic Surgery, Leiden University Medical Center, Leiden, The Netherlands; 5 Department of Rheumatology, Leiden University Medical Center, Leiden, The Netherlands; 6 Department of Clinical Epidemiology, Leiden University Medical Center, Leiden, The Netherlands

**Keywords:** Acromegaly, Arthropathy, Osteoarthritis, Management, Treatment

## Abstract

Following the description of an illustrative case of a 70-year-old female patient with longstanding active acromegaly and invalidating, progressive joint complaints, current insights regarding diagnosis, treatment, and long-term management of acromegalic arthropathy are summarized. Since clinical trials on this topic are lacking, the reported recommendations are based on extensive clinical and research experience with this clinical entity, and on established diagnostics and interventions in patients with other rheumatic diseases. The cornerstones of the management of acromegalic arthropathy remains normalization of growth hormone and insulin growth factor-1 levels. However, patients with severe or progressive acromegalic arthropathy require a multidisciplinary approach to determine adequate diagnostics and treatment options. Because of the high prevalence and invalidating character of acromegalic arthropathy, developing evidence-based effective prevention and treatment strategies, preferably by international collaboration within rare disease networks, *e.g.,* Endo-ERN, is a clear unmet need.

**Supplementary Information:**

The online version contains supplementary material available at 10.1007/s11102-024-01465-1.

## Case presentation

A 70-year-old female patient was diagnosed with acromegaly at the age of 56 years in 2008, after which she was referred to our expert center for pituitary diseases. At the time of diagnosis, she presented with temporary headaches, and optical nerve palsy, which recovered spontaneously. Retrospectively, she had a longstanding history of ‘unexplained’ complaints, including lack of energy, excessive sweating, facial changes, acral growth with the need of a larger wedding ring and shoe size, surgery for carpal tunnel syndrome (CTS, 2008), and emotional lability. Moreover, polyarticular joint complaints had been present for over 6 years, which resulted in two joint replacements (left hip (2004), and right knee (2006)), and the presence of osteoarthritic changes in both hands. Prior to the diagnosis of acromegaly, orthopedic surgeons assumed rheumatoid arthritis (RA), which was ruled out following referral to a specialized rheumatological clinic.

**Fig. 1:**
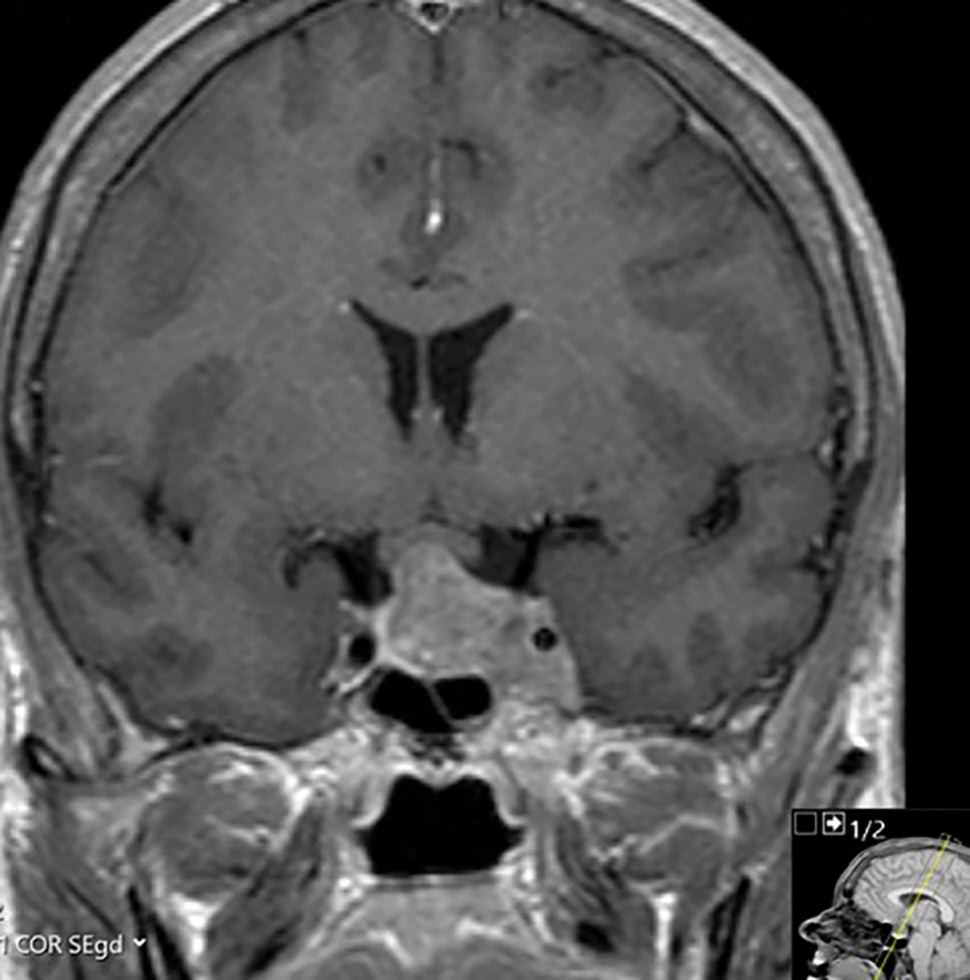
Magnetic resonance imaging of the sellar region. In 2008, the diagnostic coronal T1-weighted magnetic resonance image of the sellar region after intravenous administration of contrast revealed a macroadenoma with invasion of the left cavernous sinus and some compression of the optic chiasm

At the time of diagnosis, laboratory results showed significantly elevated growth hormone (GH; random 43.90 mU/L, and nadir following glucose suppression test 16.20 mU/L (reference range 0.00–7.25 mU/L) and insulin-like growth factor-1 (IGF-1) levels of 96.3 nmol/L (1.3 × upper limit of normal (ULN), reference range 7.0–76.0 nmol/L), and insufficient corticotropic, gonadotropic and thyrotropic axes. The pituitary MRI scan revealed a macroadenoma with invasion of the left cavernous sinus hampering total resection, as shown in Fig. 1. Cardiac examination showed left ventricular hypertrophy with mild diastolic dysfunction, and a moderate mitral and tricuspid valve insufficiency, whilst colonoscopy showed multiple colonic polyps with low grade dysplasia: all presumably complications of longstanding (undiagnosed) GH excess.

She underwent transsphenoidal surgery for tumor debulking in 2009, which could not be curative due to the cavernous sinus invasion. Additionally, pharmacological treatment with dopamine agonist (DA) and somatostatin receptor ligands (SRL) at maximum dosages was started, followed by an SRL switch to Pegvisomant because of side effects (viz*.,* alopecia). In the meantime, she received an additional right hip prosthesis (2010) because of secondary degenerative joint disease. Because of insufficient biochemical disease control with pharmacological therapy (random GH 9.49 mU/L, and IGF-1 393 nmol/L), she received pituitary radiotherapy in 2012. In 2015, Pegvisomant was stopped, after which she remained in biochemical remission to date (random GH 0.57 mU/L, IGF-1 level 9.3 nmol/L (reference range 5.1–22.0 nmol/L). Seven years after radiotherapy, she developed a GH deficiency (peak GH 5.7 mU/L using GHRH arginine test) without beneficial effects of 0.2 mg recombinant human GH (rhGH) supplementation (IGF-1 levels 14.6 nmol/L, reference range 5.1 – 22.0 nmol/L). Simultaneously, the corticotropic and thyrotropic axes were supplemented adequately. Bone mineral density (BMD) following rhGH treatment was assessed at the lumbar spine (T-score − 1.1, Z-score + 0.9; potentially unreliable due to degeneration), with hip BMD being unreliable due to joint prosthesis, and distal forearm BMD being unavailable.

**Fig. 2:**
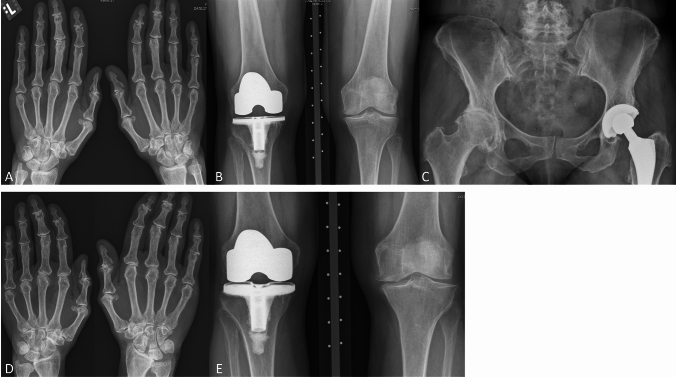
Radiographic examination of the hands, knees, and hips with follow-up. The first detailed radiographic examination of the joint complaints occurred in 2010 (**A**, **B**, **C**), with a follow-up detailed examination in 2016 (**D**, **E**). **A** Dorsovolar radiograph of the hands. Osteoarthritis of the hands, predominantly in the interphalangeal joints but also in other joints such as the MCP joints. **B** Posteroanterior radiograph of the knees. Osteoarthritis in the left knee, total knee replacement in the right knee. **C** Anteroposterior radiograph of the pelvis. Severe osteoarthritis in the right hip, total hip replacement on the left side. **D** Follow-up dorsovolar radiograph of the hands. Progression of the osteoarthritis in the hands, predominantly in the interphalangeal and MCP joints but also in other joints. **E** Follow-up posteroanterior radiograph of the knees. Progression of the osteoarthritis in the left knee

Despite achievement of biochemical remission, she had progressive, joint pain and stiffness in the hands, knees, hips, shoulders, ankles, and feet—varying in intensity depending on the joint. The joint complaints negatively influenced her daily functioning, with walking for over 15 min becoming impossible, resulting in the need for gait aids for long distances, pain medication, and physiotherapy, although she did not experience any beneficial effect of physiotherapy. Prior to retirement, she could work for 6 h a week. She was, however, able to bike and swim (non-weight-bearing exercise) without noticeable limitations. Radiographically, significant progression of structural abnormalities with end-stage arthropathy at multiple joint sites, as shown in Fig. 2, was observed. She received repetitive steroid injections in the left knee, followed by a 4th large joint prosthesis (total left knee prosthesis) in May of 2022.

## Background

Acromegaly is characterized by GH and IGF-1 excess, resulting in a plethora of clinical complaints [1–5]. Although multimodality treatment strategies—surgical pituitary adenoma resection, radiotherapy, and pharmacological treatment (*i.e.,* SRL, Pegvisomant, DAs)—are available to induce disease remission in most patients with amelioration of clinical symptoms and life expectancy, many patients suffer from (partially) irreversible, persistent, or delayed complaints due to the longstanding disease history, suboptimal biochemical control, or signs of mild GH excess [5, 6]. A major component of longstanding complaints despite disease remission are the musculoskeletal complications of acromegaly, i.e., acromegalic osteopathy with skeletal fragility, and acromegalic arthropathy [7, 8].

Acromegalic arthropathy is one of main drivers of impaired health-related quality of life (HR-QoL) [6]. Previously, clinical, and radiographic characteristics of acromegalic arthropathy in the most subjectively affected joints have been assessed (*i.e.,* hips, knees, hands, shoulders, feet, and spine joints [2, 3, 6, 9–12]). Compared to the general population, the prevalence of arthropathy is two to nine times higher—depending on the joint site—despite biochemical remission [3]. About 70% of patients in remission report joint symptoms, and virtually all patients have radiographic structural changes, which occur in a poly-articular pattern in > 95% of patients [12, 13].

The unique radiographic phenotype of patients with acromegalic arthropathy (*i.e.* primarily osteophytosis with widened joint spaces reflecting cartilage hypertrophy), is clearly different from primary osteoarthritis (OA) [14–17]. However, patients with smoldering, persistent acromegaly activity showed joint space narrowing (JSN), and more severe joint complaints more often than patients without JSN [18]. Risk factors for primary OA have been reported to also influence risk of acromegalic arthropathy (*e.g.,* age, sex, and BMI [19–22]), and there are acromegaly-specific risk factors (*e.g.* baseline IGF-1 levels, and disease duration [14–18]). Moreover, progression of acromegalic arthropathy—clinically or radiographically—has been reported for a considerable proportion of patients, independent of disease remission [10–12], with higher age, higher baseline IGF1 levels, treatment with SRL [10], and increased severity of arthropathy at baseline being the main risk factors [12].

Both acromegalic osteopathy and arthropathy are understudied to date. Recently, the current landscape of literature on the diagnosis and management of acromegalic osteopathy was summarized [7]. Despite efforts by our institution and other centers, unfortunately, little is known regarding treatment and management of acromegalic arthropathy, being a clear unmet need in the care for patients with acromegaly [13]. Although there are similarities between joint disease in acromegaly and primary OA, many features are distinct, requiring a unique diagnostic and therapeutic approach. Therefore, we aim to summarize current insights regarding the diagnosis, treatment, and long-term management of acromegalic arthropathy based on our extensive clinical and research experience, describing an illustrative case of a patient with acromegaly with progressive and invalidating joint complaints.

## Clinical assessment and diagnostics (Box 1)

### Medical history and physical examination

In this section, we summarize our findings and recommendations regarding the diagnostic options of patients with acromegalic arthropathy (Box 1). All patients with acromegaly are at risk for developing arthropathy, with specific risk factors contributing to increased risk, or increased bother in daily life, or more need for support regarding musculoskeletal disease. During general periodical visits by the endocrinologist (vide infra), a basic history assessment can be performed to screen for patients requiring specific attention for joint disease.

**Box 1:**
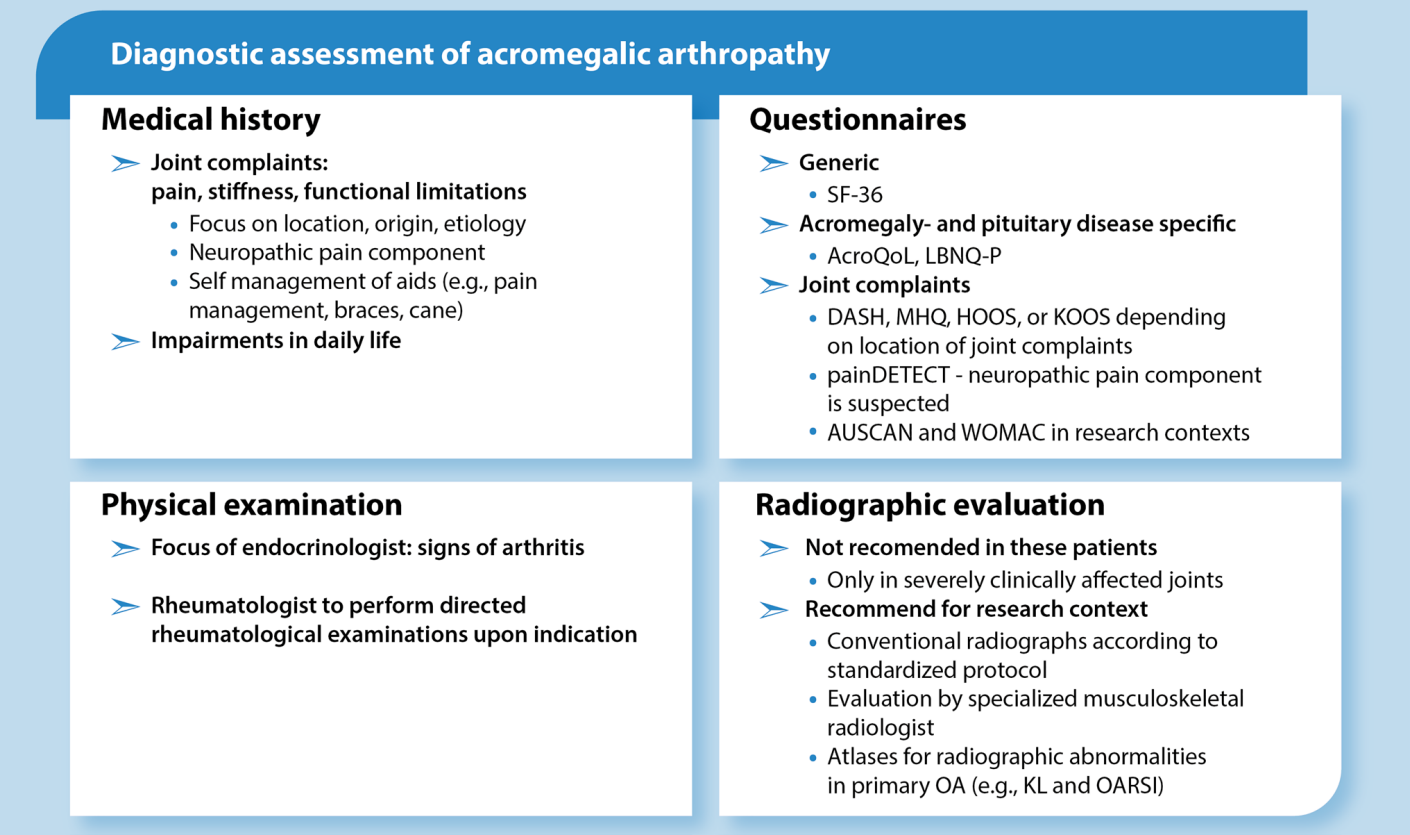
Diagnostic assessment of acromegalic arthropathy. *AcroQoL* Acromegaly quality of life questionnaire, *AUSCAN* Australian/Canadian osteoarthritis index, *DASH* Disabilities of the arm, shoulder, and hand, *HOOS* Hip disability and osteoarthritis outcome score, *KL* Kellgren and Lawrence, *KOOS* Knee injury and osteoarthritis outcome score, *LBNQ-P* Leiden Bother and needs questionnaire for pituitary patients, *MHQ* Michigan hand outcomes questionnaire, *OARSI* Osteoarthritis research society, *SF-36* Short form-36; *WOMAC* Western Ontario and McMaster Universities Osteoarthritis Index

In case of positive screening questions, musculoskeletal symptoms need to be characterized in detail by history taking and physical examination to investigate whether a differential diagnosis can be considered, how these complaints impact daily life, and if treatment is required. This history taking should focus on onset and course over time, location (mono-articular *vs* poly-articular), type of joint complaints—including nociceptive and neuropathic pain-like symptoms (*e.g.,* tingling or prickling)—and the presence of joint-related or general symptoms which could be attributed to arthritis independent of acromegaly. Additionally, limitations in activities and restrictions in societal participation should be assessed, considering hobbies, family/social life, and occupation of the patient. Furthermore, the beneficial effects of previously attempted specific interventions (*e.g.,* taping, braces, pain medication, intra-articular injections) or lifestyle changes need to be evaluated. Physical examination, including the joints, needs to be performed, with the recognition of other underlying diseases causing the joint complaints (e.g., arthritis) being the main aim of the assessment.

Based on the findings during the history taking and physical examination, patients should be referred to a rheumatologist, an orthopedic surgeon, a physiotherapist, or occupational therapist. When signs of arthritis are observed (viz., redness, warmth, swelling/hydrops, limited extension), the patient needs to be referred to a rheumatologist to exclude an underlying (immune-mediated) rheumatic disease. Notably, a relationship between acromegaly and (inflammatory) rheumatic diseases has not been proven [23].

### Use of validated questionnaires

In our value-based health care (VBHC) care path for patients with pituitary adenomas, routine use of validated questionnaires is commonplace. For patients with acromegaly, several questionnaires might be useful, including both generic and pituitary- and acromegaly-specific questionnaires. In research settings, several joint-specific questionnaires on self-reported joint symptoms have been used in patients with acromegaly [11, 12, 24–26]. These questionnaires can aid in the characterization of (the severity of) joint complaints, as well as evaluation of the impact on HR-QoL, and the specific bothers and needs prior to and after (alterations in) treatment.

In *Supplemental File 1*, generic HR-QoL questionnaires, as well as disease-specific quality of life questionnaires (i.e., Acromegaly Quality of Life Questionnaire (AcroQoL), Leiden Bother and Needs Questionnaire for patients with pituitary disease (LBNQ-Pituitary)), as well as domain-specific questionnaires assessing joint complaints are described in more detail [27–51]. At present, the LBNQ-Pituitary is the most frequently used questionnaire in clinical practice in our pituitary VBHC care path to signal whether adjustments in the management are necessary [30].

For the assessment of joint complaints in research settings, multiple joint-specific questionnaires have been used, albeit none of them validated for patients with acromegaly. Several questionnaires focused on care outcomes in patients with OA are being developed at present, which should be assessed for their usefulness in patients with acromegaly in the future.

## Radiographic assessment

### Conventional radiography

Clinically affected joints can be radiographically investigated using conventional radiographs. As is the case in primary OA, radiographic examination is not commonplace, and only indicated for patients in which a differential diagnosis needs radiological examination, or when joint replacement surgery is considered. When radiographs are indicated, a standardized radiological protocol should be used, as has been described for our center [12, 13].

Analysis of structural joint abnormalities and their severity can be performed in multiple ways. In the context of research in patients with primary OA, the semi-quantitative Kellgren and Lawrence (KL), and Osteoarthritis Research Society (OARSI) methods are the most well-known (vide infra). In the absence of validated methods for acromegalic arthropathy, both radiographic scoring methods can be used. In clinical practice, however, these scoring techniques are seldomly used.

In studies of patients with acromegaly, the most used scoring method is the semi-quantitative KL system, which is based on the evaluation of the presence of osteophytes (OP), JSN, sclerosis, and degenerative cysts in a specific joint, resulting in a composite score ranging from 0 to 4 on a 5-point Likert scale [52]. To improve scoring reliability, comparison atlases with examples of several KL scores are available for multiple joints (*e.g.,* hands, knees, hips, spine), but unfortunately not for all (*e.g.,* shoulders, feet). Another scoring method used in research is the OARSI scoring system [53, 54], evaluating individual radiographic OA characteristics separately: OP, JSN, misalignment, erosion, subchondral sclerosis, and cysts. These characteristics are scored on a 4-point Likert scale from 0 to 3. Unfortunately, the characteristic joint space widening (JSW)—hallmark of acromegalic arthropathy—is not included in any of the existing scoring methods, and might be overlooked. We therefore recommend detailed examination by an experienced musculoskeletal radiologist for clinical care purposes. For research purposes, we recommend the OARSI scoring system with the addition of (potentially automated [55–57]) JSW reading for the in-depth assessment of acromegalic arthropathy, since OA characteristics abnormalities are scored individually.

### Ultrasonography and magnetic resonance imaging

A more detailed radiographic assessment of the joint(s) might be needed. Ultrasonography is a non-invasive, high-resolution imaging technique without the need for radiation. For joint assessment, ultrasonography can detect, amongst others, effusions, and ligament and cartilage injury, which can aid in the detection of inflammatory diseases (*i.e.,* arthritis tendinitis, and bursitis). On the other hand, the role for ultrasonography in diagnosing OA is limited. Moreover, reproducibility of ultrasonography during follow-up remains highly dependent on the specialized ultrasonographer.

Magnetic resonance imaging (MRI) can be performed, *e.g.,* to exclude traumatic injury with cartilage or ligament tears, or other subtle structural changes. Previously, we have successfully and reliably performed MRI scans of the knee in patients with acromegaly, which showed the characteristic structural changes as well as decreased cartilage quality [58]. For degenerative joint disease, however, MRI scans are not indicated in the clinical diagnostic trajectory. Moreover, MRI scans are expensive, time consuming, and have limited availability.

### Categorization of patients depending on severity of arthropathy

Based on the presence of complaints, amount and location of affected joints, patients are divided into three categories: patients without joint complaints, patients with mono-articular disease (one singular joint affected) and poly-articular disease (multiple joint locations affected, e.g., hip and knee, or shoulder and hip). Moreover, patients will be divided into clinically affected, and radiologically affected, or both.

## Treatment modalities and clinical management (Box 2)

Following adequate and appropriate diagnostic strategies, we summarize our findings and recommendations regarding the treatment options and management of patients with acromegalic arthropathy (Box 4). Notably, none of the strategies outlined in this section have been formally evaluated, and are, therefore, not evidence-based in patients with acromegaly at this time. Most of the described strategies have been assessed in, and are, therefore, evidence-based for patients with primary OA.

**Box 2:**
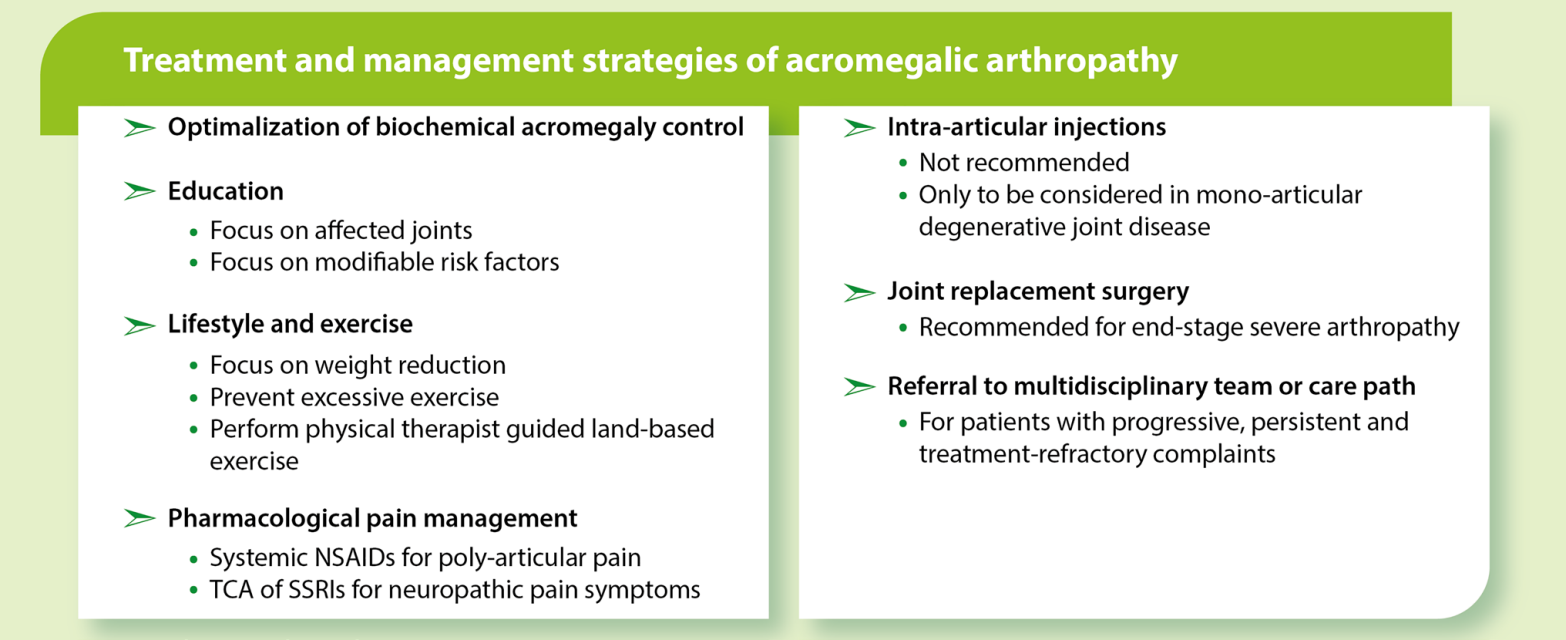
Treatment and management strategies of acromegalic arthropathy. *NSAID* non-steroidal anti-inflammatory drug, *SSRI* serotonin reuptake inhibitor, *TCA* tricyclic anti-depressant

## Management of acromegalic disease activity

The mainstay of acromegalic arthropathy management is adequate biochemical control of disease activity and symptom control [10–12, 18, 26, 59, 60]. Since controlled patients after surgical cure were reported to have improved joint outcomes compared to pharmacologically controlled patients in previous studies [12], we recommend to evaluate whether further improvement of disease control is possible, *e.g.,* whether a second surgery could be beneficial or pharmacological treatment can be intensified, also when a patient has acceptable IGF-1 levels (i.e., 1.2xULN) but still active disease symptomatology.

As mentioned, the use of pharmacological treatment (SRL, Pegvisomant and dopamine agonist combined) was associated with an increased risk of short-term progression of radiographic OA [10]. Moreover, *SRL exposure*, calculated using the dosage and duration of SRL use, was related to increased short-term radiographic OA progression, in particularly of JSN [10, 18], which was no longer observed during long-term follow-up studies [12]. Speculating, these observations could be the consequence of smoldering or residual disease activity in medically treated patients, or a direct effect of SRL itself, since SSTR are demonstrated in both joint and cartilage cells [61, 62]. As pharmacological treatment is initiated because of (persistent) disease activity, however, confounding by indication affects these observations.

## Management of joint complications

Next to the improvement of biochemical disease control, management should be focused on relief of joint complaints, and improvement of functionality. In the following paragraphs, we will discuss the different steps of the management of joint complaints in more detail [13].

### Education

Education on joint disease in acromegaly and associated modifiable risk factors is essential to improve self-management strategies [63, 64]. The most important modifiable risk factors, partially dependent on joint location, are overweight, joint injuries, and specific muscle weakness [65, 66]. Moreover, patients should be educated on how to cope with joint complaints and the therapeutic options available in general, and those applicable to their specific needs. Patient education and training of self-management strategies can occur through several ways (e.g. face-to-face meetings, group sessions, videoconferences, and telephone-based sessions, provided by e.g. specialized nurses and/or social workers/psychologists) [67]. For patients with primary OA, self-management programs are available, albeit their efficacy has not been proven [68], whereas in patients with pituitary diseases (not specifically for acromegalic arthropathy), the effectiveness of self-management programs has clearly been shown [69].

### Lifestyle and exercise

One of the most important recommendations for patients with acromegaly is maintaining a healthy lifestyle, with a healthy diet and exercise routine, and these lifestyle improvements are likely to aid in a favorable joint outcome [63, 64, 67, 70–72]. In primary OA, weight reduction in overweight and obese individuals is a well-studied and effective method to decrease strain on the weight-bearing joints. Moreover, decrease of adipose tissue mass may lead to a decrease in chronic inflammation and subsequent degenerative changes [73, 74]. Recently, a network meta-analysis on the effects of dietary interventions on the WOMAC questionnaire outcomes reported that to obtain a 50% reduction of the WOMAC score, a 25% weight reduction from baseline is necessary [75], indicating the potential of weight reduction, although the amount of weight reduction described in this study is very extreme. Notably, weight reduction should always be paired with adequate exercise routes to preserve lean body mass, and in frail patients, dietary weight management is, therefore, not recommended [76, 77].

### Rehabilitation, physical therapy, occupational therapy, and aids

Analogous with the clinical practice guidelines for polyarticular primary OA, we recommend referral to a specialized physiotherapist for guided, structured, land-based exercise programs as a core treatment for acromegalic arthropathy [63, 64, 67, 71, 78, 79], preferably early in the disease course. As an alternative, at-home exercise programs for patients with acromegaly might be beneficial as well [80]. Moreover, gait aids and mind–body exercise might be recommended in case of arthropathy of the knee and hip, based on their favorable efficacy and safety profiles in primary OA [63]. For patients with acromegaly and hand OA, physical therapy, and occupational therapy, as well as the use of aids and orthoses might results in reduced symptom severity, as mentioned in the guidelines for primary hand OA [66]. Furthermore, changes in foot shape and plantar pressure related to acromegaly, can be supported by orthopedic insoles or (semi-)orthopedic shoes in order to prevent complications and reduce potential overload and lower extremity pain [81].

### Pharmacological pain management

In patients with persistent or progressive complaints, pharmacological pain management might be of use for symptom relief. In patients with mono-articular complaints of the knee and hand, the use of topical pain-relieving creams or gels can be used [63]. For poly-articular complaints, we recommend the use of non-selective NSAIDs (*e.g.,* naproxen), or selective COX-2 inhibitors (*e.g.,* etericoxib, or rofecoxib) as a first-line treatment, but only on demand for short periods of time, since there is no evidence for pain relief with chronic use in combination with an unfavorable toxicity profile.

Although most patients with acromegaly suffer from nociceptive joint pain, a minority of patients (*i.e.*, around 25%) present with (possible) neuropathic pain-like symptoms [24]. Since neuropathic pain requires a different pharmacological approach, neuropathic pain-like symptoms need to be recognized early. Patients with neuropathic pain-like symptoms could benefit from treatment with specific neuropathic pain medication, including tricyclic antidepressants (*e.g.,* amitriptyline), anti-epileptic drugs (*e.g.,* pregabalin/gabapentin), or a serotonin–norepinephrine reuptake inhibitor (*e.g.,* duloxetine [82–84]).

### Intra-articular injections

Intra-articular injections with corticosteroids or hyaluronic acid are expected to be of limited value because of the poly-articular nature of acromegalic arthropathy in most patients, and are, therefore, not recommended [64, 66, 85]. However, in the rare case of mono-articular joint complaints with signs of an inflammatory origin, intra-articular corticosteroids can be considered.

### Joint replacement surgery

In case of persistent invalidating end-stage arthropathy, joint replacement surgery, or other articular surgical interventions, can be considered as a last resort [86]. Orthopedic surgery should be considered in patients with pain, limited mobility, and functional disabilities, when conservative treatment strategies have failed to provide symptomatic relief or improvement of quality of life. Conventional radiographs are used to assess the severity of radiographic OA. Joint replacement surgeries should be planned following expert consultation with an orthopedic surgeon. From a translational research perspective, the joint tissues and biological materials removed during joint replacement surgery (*i.e.,* ligaments, cartilage, and bone) could provide us with valuable insights on the underlying mechanisms of joint disease in acromegaly. In the future, a biobank for joint tissues, potentially in cooperation with the national and European scientific societies and the European Reference Network for endocrine conditions (Endo-ERN) might be considered.

### Multidisciplinary team referral and intensive care path for complex patients

Patients with acromegaly with persistent or progressive arthropathy could be referred to an expert center with a multi-disciplinary team (MDT) consisting of specialized endocrinologists, rheumatologists, radiologists, orthopedic surgeons, pain specialists, rehabilitation physicians, and psychologists for expert opinion. During MDT meetings, the appropriate actions to be taken for individual patients need to be discussed. In our center, an intensive 6-week multi-disciplinary care path exists for patients with common rheumatological diseases (*e.g.*, RA), including weekly or twice-weekly sessions with a rheumatologist, physiotherapist, occupational therapist, and psychologist. This intensive care path encompasses detailed diagnostics and therapeutic strategies, with the aim for optimal management of joint disease. Following this 6-week care path, patients will be referred back to their treating physician with an individualized management advice. This care path can serve as an example for the creation of a VBHC care path for patients with acromegalic arthropathy, highlighting the improvement of care for rare diseases though regular care for more common diseases.

## Discussion and future perspectives

In the present overview, we highlighted the recommended diagnostic and management options for patients with acromegalic arthropathy based on extensive clinical expertise, the current literature from acromegaly arthropathy, and guidelines for patients with primary OA. In absence of clinical trials focusing on acromegalic arthropathy, all diagnostic and treatment strategies mentioned are not (yet) evidence-based, highlighting the need for studies on this topic.

First, the necessity of a knowledgeable and experienced MDT needs to be stressed for the adequate diagnostic and therapeutic strategies, as well as the management of acromegalic arthropathy. Organizing care within expert centers—functioning as a pituitary center of excellence (PTCOE)—with MDTs with additional expertise in joint disease on long-term complications of acromegaly (including acromegalic arthropathy in patients with acromegaly) is preferred [87–90].

As the diagnostic age for acromegaly and primary OA is ≥ 50 years, and acromegalic arthropathy has been associated with older age, the outlined recommendations also apply for elderly patients [91, 92]. Regardless of acromegaly, management of elderly patients should focus on maintenance or improvement of muscle mass and function and decreasing the risk of falling, and for potential surgeries or other interventions, the health status, comorbidities, as well as the chances to achieve the surgical goal and potential risks, need to be carefully considered [63, 64, 67, 71, 78, 79].

The recommended strategies focus on joint complaints. Although these strategies aim for optimal management, patients might suffer from persistent residual complaints with a negative impact on their daily functioning and quality of life [5–8]. Patient education programs and/or individual counseling by a psychologist can support patients with coping with these complaints and their effect on daily life [69].

Due to the direct and indirect effects of GH and IGF-1 on bone, patients with acromegaly can suffer from both arthropathy and osteopathy. Acromegalic osteopathy is characterized by a decrease in bone quality resulting in skeletal fragility and increased fracture risk, despite normal BMD measured by DXA scans [5, 7, 8, 93]. As for arthropathy, adequate control of disease activity is the cornerstone of treatment of acromegalic osteopathy [5, 7, 8, 93]. Additionally, maintaining a eugonadal state and adequate vitamin D levels may improve bone quality [5, 7, 8, 93]. Notably, bone acting drugs have been shown to decrease incident vertebral fractures in patients with active disease [94]. Moreover, the effects of bone-acting drugs in patients with acromegaly with disease control might play an additional role—as in patients with osteoporosis [95–97], although studies are lacking.

Notably, not all therapeutic strategies are without risk, and the risks and benefits of joint replacement surgeries especially must be weighed on an individual patient level whilst considering their overall (medical) situation. Because of the concomitant osteopathy [8, 93], joint implants theoretically might take longer to adhere to the bone (in the case of uncemented prostheses), or might results in fragility fractures of the weakened bone next to the implant, or other forms of aseptic loosening or dislocation of the implant, as reported in the sole small cohort study in patients with acromegaly [98]. Although these complications have not occurred in patients with acromegalic arthropathy following joint replacement surgery in our expert center, the potential risks in this specific patient group must be considered. Expert knowledge on joint replacement surgeries in patients with acromegaly should be combined nationally or internationally, as these surgeries are not performed by one dedicated orthopedic surgeon for all patients.

Several therapeutic or management strategies have not been mentioned prior, one of which being dietary supplements, vitamins, and minerals. However, in our experience, patients with acromegaly do use several supplements to increase bone and cartilage health, of which glucosamine was the most frequently used. In literature, the effects of these supplements on bone and cartilage health in primary OA is not recommend [99–101].

All therapeutic strategies described in the previous sections focus on symptomatic relief in patients with acromegalic arthropathy, whereas cure or prevention of acromegalic arthropathy is not attainable to date. The potential beneficial effect of education, weight loss, or physical therapy prior for the prevention of joint complaints needs to be assessed. In the future, more detailed characterization of the different phenotypes of acromegalic arthropathy – presence of OP, JSN or JSW—will aid in the discovery of new treatment strategies, as patients exhibit different radiographic phenotypes: an OA-like phenotype, or a characteristically acromegalic phenotype [18, 58]. For these phenotypes, the impact of GH excess might play a different, yet-to-be-elucidated role. Moreover, prospective studies should focus on the natural history and disease course in these patients using standardized and protocolized outcome measures. Moreover, translational studies using tissue samples (*e.g.,* cartilage and bone resected during joint replacement surgery) should focus on the underlying mechanisms, and, thereby, potential drug targets, of the development of acromegalic arthropathy.

In conclusion, recommended diagnostic, therapeutic, and management strategies, as well as the need for a multidisciplinary approach, of patients with acromegalic arthropathy have been summarized. To date, treatment options are solely symptomatic, not curative as in OA and pragmatic and not evidence based. Therefore, future studies should focus on effective prevention and treatment strategies of arthropathy in acromegaly, preferably within international collaborations to join forces for this rare condition, as this is a great unmet need for patients with acromegaly.

## Supplementary Information

Below is the link to the electronic supplementary material.

Supplementary file1 (DOCX 54 KB)

## Data Availability

No datasets were generated or analysed during the current study.
