# Supplementary material for: Approach to the patient with controlled acromegaly and acromegalic arthropathy: clinical diagnosis and management
Source: Pituitary. 2024 Nov 1;27(6):824–36. doi: 10.1007/s11102-024-01465-1 (PMC11632006; doi:10.1007/s11102-024-01465-1)
Supplement: Supplementary file 1 — Supplementary file1 (DOCX 54 KB) [file 11102_2024_1465_MOESM1_ESM.docx]

# Approach to the patient with controlled acromegaly and acromegalic arthropathy: Clinical diagnosis and management

Iris C.M. Pelsma^1^, Herman M. Kroon^2^, Cornelie D. Andela^1,3^, Enrike M.J. van der Linden^4^, Margreet Kloppenburg^5,6^, Nienke R. Biermasz^1^, Kim M.J.A. Claessen^1^

**Author affiliations**

1. Dept. of Medicine, Division of Endocrinology, and Center for Endocrine Tumors Leiden, Leiden University Medical Center, Leiden, the Netherlands
2. Dept. of Radiology, Leiden University Medical Center, Leiden, the Netherlands
3. Basalt Rehabilitation Center, The Hague, the Netherlands
4. Dept. of Orthopedic Surgery, Leiden University Medical Center, Leiden, the Netherlands
5. Dept. of Rheumatology, Leiden University Medical Center, Leiden, the Netherlands
6. Dept. of Clinical Epidemiology, Leiden University Medical Center, Leiden, the Netherlands

**Corresponding author**

Iris C.M. Pelsma
Department of Medicine, Division of Endocrinology
Leiden University Medical Center,
Albinusdreef 2,
2333 ZA Leiden, The Netherlands
Tel: +31 71 526 8172
Email: [I.C.M.Pelsma@lumc.nl](mailto:I.C.M.Pelsma@lumc.nl)

**Short title (max. 50 characters with spaces):** Management of acromegalic arthropathy

## Supplemental File 1

### Use of validated questionnaires

In our value-based health care (VBHC) care path for patients with pituitary adenomas, routine use of validated questionnaires is commonplace. For patients with acromegaly, several questionnaires might be useful, including both general and pituitary- and acromegaly-specific questionnaires. In research settings, several joint-specific questionnaires on self-reported joint symptoms have been used in patients with acromegaly (1-5). These questionnaires can aid in the characterization of (the severity of) joint complaints, as well as evaluation of the impact on HR-QoL, and the specific bothers and needs prior to and after (alterations in) treatment. Below, the questionnaires used are described in more detail.

#### General health-related quality of life

The validated *Short Form-36* (SF-36) is used to assess patients’ general HR-QoL during the previous 30 days. Thirty-six questions accompanied by standardized response choices measure eight HR-QoL domains. Total scores range from 0 to 100 for all domains, with higher scores reflecting higher HR-QoL (6, 7).

#### Acromegaly-specific quality of life

The *Acromegaly Quality of Life Questionnaire* (AcroQoL) is an acromegaly-specific questionnaire assessing HR-QoL (8), consisting of 22 statements being rated on a 5-point Likert scale. Responses given regard the frequency of occurrence (always – never), and the degree of item agreement (completely agree – completely disagree), with total scores ranging from 0-100, with lower scores indicating more impaired HR-QoL. Statements relevant for joint disease include: ‘My legs feel weak’, ‘Some parts of my body are too big’, ‘I have problems doing things with my hands’, ‘My joints ache’, and ‘I feel weak’.

#### Perceived bother and needs for support in patients with pituitary disease

The *Leiden Bother and Needs Questionnaire for pituitary patients* (LBNQ-Pituitary) is a disease-specific questionnaire, which was developed in our center based on focus groups among patients with pituitary diseases. This questionnaire consists of 26 items divided into five subscales: mood problems, negative illness perceptions, issues in sexual functioning, physical and cognitive complaints, and issues in social functioning (9). All subscales are scored on calculated index scores (range 0–100), with higher scores indicating greater bother by the disease, or higher needs for support for the disease. At present, this is the most frequently used questionnaire in clinical practice in our pituitary VBHC care path to signal whether adjustments in the management are necessary.

#### Self-reported joint complaints

Based on the location of the joint complaints, specific validated questionnaires can be used to further characterize self-reported joint symptoms. All these questionnaires have a numeric outcome, of which the significance and interpretation is stated below. These questionnaires have been developed for the assessment of symptoms in primary OA, and, therefore, their use in clinical practice in patients with acromegaly is limited.

For symptoms of the upper limbs, the *Disabilities of the Arm,* *Shoulder, and Hand* (DASH) assesses physical function, and symptoms of the upper limb during the previous 7 days, using 30 questions with a 5-point Likert scale (10-13). Higher total DASH scores (range 0 – 100) signify greater disability. The *Michigan Hand Outcomes Questionnaire* (MHQ) assesses hand complaints in the past week using 57 questions spanning 6 domains scored on a 5-point Likert scale: overall hand function, activities of daily living, work performance, aesthetics, patient satisfaction, and pain (14, 15). The former 5 MHQ domains (range 0 – 100) are interpreted as higher scores indicating less disability, whereas the pain domain (range 0 – 100) needs to be inversed (since a score of 0 indicates no pain), with total MHQ score being the mean of all 6 domains.

With respect to the lower limbs, the *Hip disability and osteoarthritis outcome score* (HOOS) assesses hip disability using 40 questions, scored on a 5-point Likert scale, summing up to five subscales: pain, other symptoms, function in daily living, function in sport and recreation, and hip-related QoL (16, 17). Each subscale is scored on a range of 0-100 with higher scores indicating less complaints. The *Knee injury and Osteoarthritis Outcome Score* (KOOS) assesses knee complaints during the last week (18, 19). The KOOS consists of 42 items scored on a 5-point Likert-scale spanning the same subscales as the HOOS questionnaire, and the scores being calculated similarly (range 0–100; higher scores indicating less complaints). For research purposes, the *Australian/Canadian Osteoarthritis Index* (AUSCAN) and the *Western Ontario and McMaster Universities Osteoarthritis Index* (WOMAC) questionnaires have been used in patients with acromegaly for the assessment of hand and lower limb symptoms (20, 21). Notably, AUSCAN and WOMAC are not widely available, hampering the use by several centers.

#### Neuropathic pain component

In patients with signs of neuropathic-like pain symptoms, the *painDETECT questionnaire* can be used to assess aspects of neuropathic pain, including pain localization and pain course over time (22-27). Based on the total painDETECT scores (range 0 – 35 based on 7 questions using a 6-point Likert scale from 0 to 5), patients can be divided into three groups, reflecting the probability of neuropathic-like pain symptoms: unlikely, total score ≤12; indeterminate, total score 13-18; likely, total score ≥19 (28-30).

### References

1. Claessen KM, Ramautar SR, Pereira AM, Romijn JA, Kroon HM, Kloppenburg M, et al. Increased clinical symptoms of acromegalic arthropathy in patients with long-term disease control: a prospective follow-up study. Pituitary. 2014;17(1):44-52.

2. Pelsma ICM, Biermasz NR, van Furth WR, Pereira AM, Kroon HM, Kloppenburg M, et al. Progression of acromegalic arthropathy in long-term controlled acromegaly patients: 9 years of longitudinal follow-up. J Clin Endocrinol Metab. 2020.

3. Pelsma ICM, van Trigt VR, Kroon HM, Pereira AM, van der Meulen C, Kloppenburg M, et al. Low prevalence of neuropathic-like pain symptoms in long-term controlled acromegaly. Pituitary. 2021;In press.

4. Wassenaar M, Biermasz N, Kloppenburg M, van der Klaauw A, Tiemensma J, Smit J, et al. Clinical osteoarthritis predicts physical and psychological QoL in acromegaly patients. Growth Hormone & IGF Research. 2010;20:226-33.

5. Pelsma ICM, Kroon HM, van Trigt VR, Pereira AM, Kloppenburg M, Biermasz NR, et al. Clinical and radiographic assessment of peripheral joints in controlled acromegaly. Pituitary. 2022;25(4):622-35.

6. Aaronson NK, Muller M, Cohen PD, Essink-Bot ML, Fekkes M, Sanderman R, et al. Translation, validation, and norming of the Dutch language version of the SF-36 Health Survey in community and chronic disease populations. J Clin Epidemiol. 1998;51(11):1055-68.

7. Brazier JE, Harper R, Jones NM, O'Cathain A, Thomas KJ, Usherwood T, et al. Validating the SF-36 health survey questionnaire: new outcome measure for primary care. BMJ. 1992;305(6846):160-4.

8. Webb SM, Prieto L, Badia X, Albareda M, Catala M, Gaztambide S, et al. Acromegaly Quality of Life Questionnaire (ACROQOL) a new health-related quality of life questionnaire for patients with acromegaly: development and psychometric properties. Clin Endocrinol (Oxf). 2002;57(2):251-8.

9. Andela CD, Scharloo M, Ramondt S, Tiemensma J, Husson O, Llahana S, et al. The development and validation of the Leiden Bother and Needs Questionnaire for patients with pituitary disease: the LBNQ-Pituitary. Pituitary. 2016;19(3):293-302.

10. Khan WS, Jain R, Dillon B, Clarke L, Fehily M, Ravenscroft M. The 'M2 DASH'-Manchester-modified Disabilities of Arm Shoulder and Hand score. Hand (N Y). 2008;3(3):240-4.

11. Beaton DE, Katz JN, Fossel AH, Wright JG, Tarasuk V, Bombardier C. Measuring the whole or the parts? Validity, reliability, and responsiveness of the Disabilities of the Arm, Shoulder and Hand outcome measure in different regions of the upper extremity. J Hand Ther. 2001;14(2):128-46.

12. Solway S BD, McConnell S, Bombardier C. The DASH outcome measure user’s manual. 2002. Contract No.: 2nd ed. .

13. Veehof MM, Sleegers EJ, van Veldhoven NH, Schuurman AH, van Meeteren NL. Psychometric qualities of the Dutch language version of the Disabilities of the Arm, Shoulder, and Hand questionnaire (DASH-DLV). J Hand Ther. 2002;15(4):347-54.

14. Chung KC, Pillsbury MS, Walters MR, Hayward RA. Reliability and validity testing of the Michigan Hand Outcomes Questionnaire. J Hand Surg Am. 1998;23(4):575-87.

15. Nolte MT, Shauver MJ, Chung KC. Normative Values of the Michigan Hand Outcomes Questionnaire for Patients with and without Hand Conditions. Plast Reconstr Surg. 2017;140(3):425e-33e.

16. Klassbo M, Larsson E, Mannevik E. Hip disability and osteoarthritis outcome score. An extension of the Western Ontario and McMaster Universities Osteoarthritis Index. Scand J Rheumatol. 2003;32(1):46-51.

17. Nilsdotter AK, Lohmander LS, Klassbo M, Roos EM. Hip disability and osteoarthritis outcome score (HOOS)--validity and responsiveness in total hip replacement. BMC Musculoskelet Disord. 2003;4:10.

18. Roos EM, Lohmander LS. The Knee injury and Osteoarthritis Outcome Score (KOOS): from joint injury to osteoarthritis. Health Qual Life Outcomes. 2003;1:64.

19. Roos EM, Roos HP, Lohmander LS, Ekdahl C, Beynnon BD. Knee Injury and Osteoarthritis Outcome Score (KOOS)--development of a self-administered outcome measure. J Orthop Sports Phys Ther. 1998;28(2):88-96.

20. Bellamy N, Campbell J, Haraoui B, Gerecz-Simon E, Buchbinder R, Hobby K, et al. Clinimetric properties of the AUSCAN Osteoarthritis Hand Index: an evaluation of reliability, validity and responsiveness. Osteoarthritis Cartilage. 2002;10(11):863-9.

21. Bellamy N, Buchanan WW, Goldsmith CH, Campbell J, Stitt LW. Validation study of WOMAC: a health status instrument for measuring clinically important patient relevant outcomes to antirheumatic drug therapy in patients with osteoarthritis of the hip or knee. J Rheumatol. 1988;15(12):1833-40.

22. Rienstra W, Blikman T, Mensink FB, van Raay JJ, Dijkstra B, Bulstra SK, et al. The Modified painDETECT Questionnaire for Patients with Hip or Knee Osteoarthritis: Translation into Dutch, Cross-Cultural Adaptation and Reliability Assessment. PLoS One. 2015;10(12):e0146117.

23. De Andrés J, Pérez-Cajaraville J, Lopez-Alarcón MD, López-Millán JM, Margarit C, Rodrigo-Royo MD, et al. Cultural adaptation and validation of the painDETECT scale into Spanish. Clin J Pain. 2012;28(3):243-53.

24. Alkan H, Ardic F, Erdogan C, Sahin F, Sarsan A, Findikoglu G. Turkish version of the painDETECT questionnaire in the assessment of neuropathic pain: a validity and reliability study. Pain Med. 2013;14(12):1933-43.

25. Matsubayashi Y, Takeshita K, Sumitani M, Oshima Y, Tonosu J, Kato S, et al. Validity and reliability of the Japanese version of the painDETECT questionnaire: a multicenter observational study. PLoS One. 2013;8(9):e68013.

26. Gudala K, Ghai B, Bansal D. Neuropathic Pain Assessment with the PainDETECT Questionnaire: Cross-Cultural Adaptation and Psychometric Evaluation to Hindi. Pain Pract. 2017;17(8):1042-9.

27. Sung JK, Choi JH, Jeong J, Kim WJ, Lee DJ, Lee SC, et al. Korean Version of the painDETECT Questionnaire: A Study for Cultural Adaptation and Validation. Pain Pract. 2017;17(4):494-504.

28. Cappelleri JC, Koduru V, Bienen EJ, Sadosky A. A cross-sectional study examining the psychometric properties of the painDETECT measure in neuropathic pain. J Pain Res. 2015;8:159-67.

29. Freynhagen R, Tölle TR, Gockel U, Baron R. The painDETECT project - far more than a screening tool on neuropathic pain. Curr Med Res Opin. 2016;32(6):1033-57.

30. Freynhagen R, Baron R, Gockel U, Tölle TR. painDETECT: a new screening questionnaire to identify neuropathic components in patients with back pain. Curr Med Res Opin. 2006;22(10):1911-20.
